# Control of Photoinduced Ligand Detachment from Quantum Dot Surfaces Using Nanocrystal Architecture

**DOI:** 10.1021/acs.jpclett.6c01698

**Published:** 2026-08-10

**Authors:** McKenna N. Grega, Jacob A. Cho, Robert A. Brown, John B. Asbury

**Affiliations:** Department of Chemistry, 8082 The Pennsylvania State University, University Park, Pennsylvania 16802, United States

## Abstract

We investigated the influence that nanocrystal architecture has on the extent to which colloidal quantum dots undergo photoinduced ligand detachment. We took advantage of the ability of electrons to delocalize throughout both the core and shell of CdSe/CdS core/shell nanostructures to modulate the surface electron density of a series of colloidal quantum dots. Then, we used infrared transient absorption spectroscopy to monitor the ligand photodetachment process on the surfaces of quantum dots with varying shell thicknesses. CdSe core-only and CdSe/CdS quantum dots with thin CdS shells exhibit little photoinduced ligand detachment in the solid film environment. In contrast, CdSe/CdS core/shell nanostructures with thicker CdS shells that localize surface electron density in their excited states undergo extensive ligand photodetachment even in solid films. Our findings suggest that core/shell architectures with tuned shell thicknesses could enhance the extent of photoinduced ligand detachment and could increase the photocatalytic activity of colloidal quantum dots.

## Graphical abstract

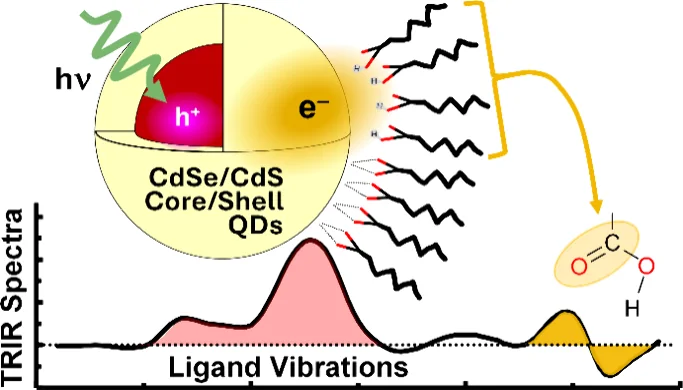

Molecular dynamics at the nanocrystal-ligand boundaries of colloidal quantum dots (QDs) can have a strong influence on the rate and yield of charge transfer processes and photocatalytic transformations at their surfaces.^1–15^ For example, the dynamics of ligands attached to QDs are often coupled to charge dynamics at their surfaces,^16–19^ which can influence their photocatalytic activity.^20–23^ CdSe nanorods decorated with Pt nanoparticles and passivated by mercaptopropionic acid were shown to undergo hole localization at their surfaces, which extended their charge separation lifetime and enhanced their photocatalytic quantum yields.^20–22^ Furthermore, the permeability of the ligand shells of QDs can influence the rates of forward and back charge transfer processes^21,24–39^ as well as the ability of charge acceptors to undergo charge transfer within the excited state lifetimes of the QDs.^39^

Despite the central role that charge and molecular dynamics can have in mediating the energy and charge transfer processes at QD surfaces, it has been challenging to identify the specific types of charge carriers and excited state surface chemistries that give rise to this behavior. Nonetheless, a number of studies have provided insights about such charge-ligand interactions. For example, the injection of excess electrons into CdSe QDs led to the displacement of X-type ligands such as carboxylates as a result of the need to maintain charge balance.^19^ Steady-state and time-resolved Raman spectroscopy permitted the investigation of the coupling of ligand vibrational modes with electronic states of QDs.^40,41^ Ligands such as thiols have frontier orbitals that can mix with electronic states of CdSe and related metal chalcogenides,^19,42^ which enhances the coupling of ligand vibrations with electronic states of the QDs and accelerates nonradiative exciton decay pathways,^43–45^ leading to quenched photoluminescence.

Recent time-resolved infrared (TRIR) spectroscopy studies of the excited state surface chemistry of QDs revealed that ligands can transiently photodetach from their surfaces, temporarily creating a more permeable ligand shell.^16–18^ Systematic studies of this process correlated with X-type ligand density on the QDs revealed that the photodetachment process is driven by interactions of ligands with surface electron density in the excited states of the QDs.^46^ These findings suggested the possibility of varying the molecular dynamics of ligands on QDs by controlling the type of charge carriers that can localize at their surfaces. For example, investigations of various nanocrystal architectures revealed that CdSe/CdS core/shell structures tend to confine holes to the cores of the nanocrystals while allowing electrons to delocalize throughout both the core and shell of the nanocrystals.^47^ Such nanocrystals can exhibit both type-I or type-II heterojunction behavior wherein electron density can be either delocalized into the CdS phase or charge separated entirely.^48,49^ In this work, we did not attempt to distinguish these behaviors but rather note that both behaviors led to partitioning of electron density to the surfaces of the CdSe/CdS nanostructures. This is the property that we utilized in this work.

Motivated by these observations, we hypothesized that it may be possible to control the extent of photoinduced ligand detachment from colloidal QD surfaces by selection of the appropriate nanocrystal architecture. To test this hypothesis, we synthesized CdSe/CdS core/shell QDs with varying shell thicknesses and examined the corresponding influences on the excited state lifetimes and molecular dynamics at their nanocrystal-ligand boundaries. In this work, we examined these excited state dynamics in solid QD films (no solvent) to simplify the spectroscopy. Our findings reveal that CdSe core-only QDs (no shells) exhibit hole-associated Fano-type transient vibrational features that result from coupling of ligand carboxylate anchoring groups to surface localized hole density in analogy to prior studies.^18^ Similarly, CdSe/CdS core/shell QDs with thin CdS shells retained their hole-associated carboxylate features.

In contrast, CdSe/CdS core/shell QDs with thicker shells insulated the nanocrystal-ligand boundary from hole density, which suppressed hole-associated carboxylate features in the excited states of the nanocrystals. The preferential enhancement of surface electron density in the thicker shells of the nanocrystals perturbed their surfaces to such an extent that ligands photodetached and became protonated despite the QDs being arranged in dense solid films. The newly protonated carboxylic acid groups however remained close to the QD surfaces and adopted weakly coordinated geometries similar to those identified recently by NMR studies.^50–52^ Taken together, our findings revealed that nanocrystal architectures that separate electron and hole densities in different regions of the nanostructures for longer periods of time may be used to systematically enhance the density of ligands that photodetach from QD surfaces. This suggests that nanocrystal architectures such as core/shell QDs or dot-in-rod structures can be used to transiently create more permeable ligand shells around the nanocrystals that are passivated by X-type ligands. A logical inference from this work is that such transient increase in ligand shell permeability could be used in future studies to promote photoinduced charge transfer processes during photocatalytic reactions in solution while still allowing the QDs to be fully coordinated between excitation events.

To establish these findings, we examined the excited state surface chemistry of CdSe and CdSe/CdS core/shell QDs to determine whether their photoinduced ligand dynamics are influenced by the type of charge carrier (electrons versus holes) that localize at their surfaces. We synthesized a series of CdSe QDs with varying core/shell architectures as described in Section S1 and illustrated in Figure S1. We then examined the influence that the surface charge density in the excited states of CdSe core-only and CdSe/CdS core/shell nanocrystals had on the photoinduced ligand detachment processes at their nanocrystal-ligand boundaries using TRIR spectroscopy (Figure 1A).

**Figure 1:**
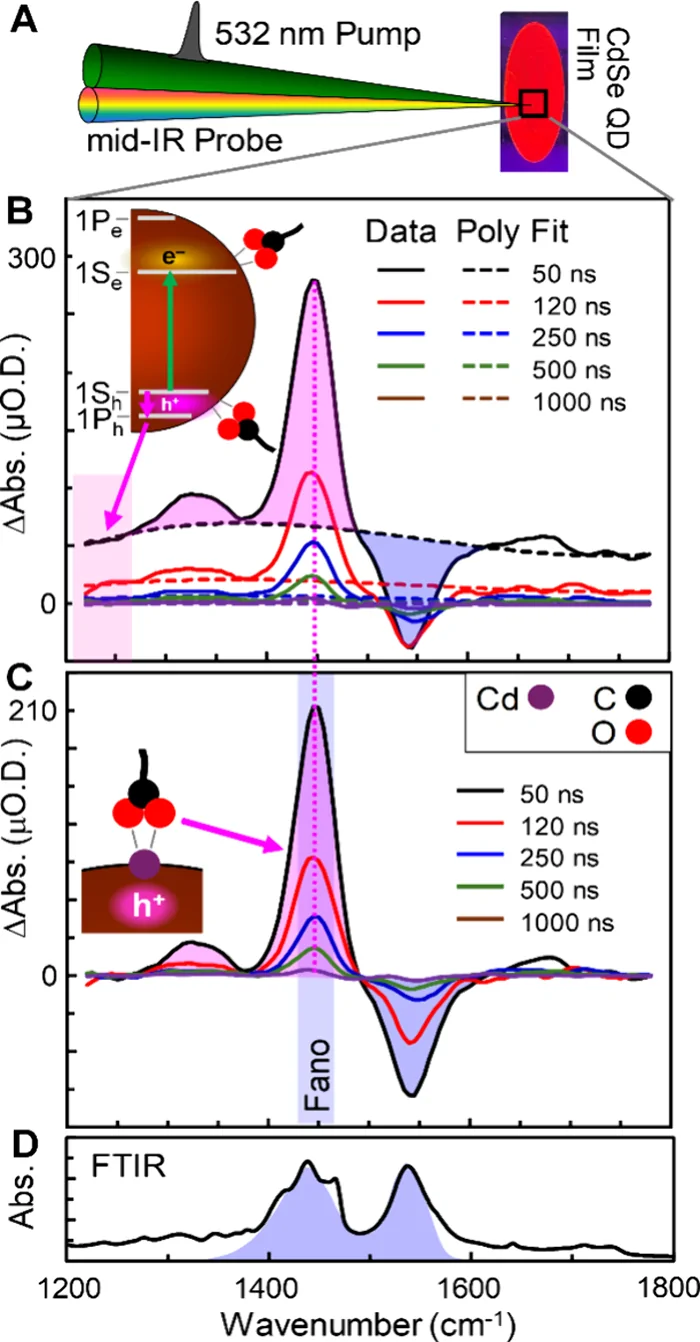
(A) Time-resolved infrared (TRIR) spectroscopy was used to examine the excited state surface chemistry of stearate ligands attached to CdSe core-only QDs in solid films. (B) TRIR spectra of a CdSe QD film (Data) following 532 nm excitation. The transient vibrational features of the ligands were superimposed on broad electronic absorption offsets indicated by dotted lines (Poly Fit). The pink shaded box marks the frequencies where the decay kinetics of the electronic absorption offset were measured. (C) Transient vibrational spectra obtained by subtraction of the broad electronic offset from the measured TRIR spectra at each time delay. The pink and blue shaded peaks highlight ‘hole associated’ carboxylate Fano-type vibrational features of the ligands that are similarly highlighted in panel B. The vertical blue shading marks frequencies where the kinetics of the Fano-type resonance were measured after subtraction of the electronic offsets. (D) The ground state carboxylate vibrational absorbance features appeared in the FTIR spectrum of the CdSe QD film.

Figure 1B presents TRIR spectra of CdSe core-only QDs passivated by steric acid ligands and deposited as a dense solid film following optical excitation by a 532 nm excitation pulse. This sample was included in this study as a control to show continuity with our prior measurements of the excited state surface chemistry of CdSe QDs measured in solid films^18^ and in the solution environment.^46^ The film thickness, optical absorption at 532 nm (Figure S1A) and excitation intensity of 20 μJ/cm^2^ were chosen so that approximately 1 out of every 10 QDs in the sample absorbed a pump photon. The excitation wavelength was also tuned to the excitonic bandgap of the QDs to minimize the excess energy deposited into the nanocrystals in their excitonic excited states. Similar to our prior practice, the TRIR spectra represented averages over time ranges that varied according to the time delay. For example, the spectra labeled 50, 120, and 250 ns represented averages of spectra measured every 2 ns between 50 and 70 ns, 120–150 ns, and 250–300 ns, respectively. Table S1 lists the full time ranges that were averaged to produce the spectra in Figure 1B.

The TRIR spectra of the CdSe core-only QDs exhibit three distinct spectral components that were characteristic of transient infrared absorption features of QDs in their excited excitonic states. Narrow vibrational features of ligands on the QDs that were perturbed by interaction with the excitonic excited states appeared in the spectra superimposed on a broad absorption offset as indicated by pink and blue shading. Previous measurements of CdSe QDs with similar excitonic bandgaps revealed that the electronic absorption offset arose from the 1S-1P intraband transition of holes.^18^ The 1S-1P intraband transition of electrons of similarly sized CdSe QDs appeared at higher transition energies around 0.5 eV. To isolate the transient vibrational features of the ligands, we fit the electronic absorption offsets with third-order polynomial functions (dotted curves in Figure 1B) and subtracted them from each TRIR spectrum. The transient vibrational spectra resulting from this subtraction appear in Figure 1C using the same color scheme for each time delay.

The same pink and blue shading of the transient vibrational features was used in Figure 1C for clarity to show that the shapes of the vibrational features could be easily distinguished from the shapes of the third-order polynomial fit functions. These vibrational features closely resembled prior measurements of the vibrational features of CdSe QDs passivated by steric acid also in solid films.^18^ Prior studies of CdSe QDs revealed that undercoordinated Se atoms led to localization of hole density at their surfaces.^53^ Comparison of the positive- and negative-going transient vibrational features to the FTIR absorbance spectrum of the sample (Figure 1D) revealed that they corresponded to the symmetric and antisymmetric carboxylate stretch vibrational modes of surface bonded carboxylate groups on CdSe QDs.^54,55^ These features have been assigned to ‘hole-associated’ carboxylate features,^17^ which are Fano-type resonances^56,57^ resulting from carboxylate anchoring groups that interacted with localized hole density on the CdSe QD surfaces. Similar hole-associated carboxylate features were also observed in CdSe QDs in solution with dense ligand shells that suppressed the localization of electron density at their surface Cd atoms.^46^ We note that the as-synthesized ligand density of the CdSe QDs examined here should be comparable to the 3.0:1 oleic acid-to-Cd ratio QDs examined in our recent work.^46^ However, the ligand shell density of the CdSe and CdSe/CdS QDs examined here should be higher than the comparable QDs from the earlier solution studies because the QDs examined here were cast as solid films. Therefore, the attached ligands did not need to partition between the bulk solution and the QD surfaces in this study.

To explore the influence that a thin CdS shell had on the excited state surface chemistry of the QDs, we synthesized 2 ML CdSe/CdS core/shell nanocrystals following literature procedures^58^ as described in Section S1. We verified that the CdS shells conformally coated the CdSe cores by comparing the quenching effectiveness of a 17 mM concentration of dodecanethiol ligands that were introduced into the 2 ML CdSe/CdS QDs dissolved in hexane solutions. As shown in Figure S2, introduction of dodecanethiol into CdSe core-only solutions led to complete quenching of the excitonic emission as has been observed previously.^43–45^ The interaction of thiols with CdSe surfaces led to the formation of midgap states that split excitons and prevented their radiative relaxation. However, when the same concentration of thiol ligands were introduced into the 2 ML CdSe/CdS QD solutions, their excitonic emission changed negligibly, indicating that the CdS shells conformally coated the CdSe cores. The shells effectively insulated the excitonic excited states from the thiol-related midgap states. The excitonic emission of 6 ML CdSe/CdS core/shell QDs was also preserved in the presence of the same concentration of thiol ligands.

Figure 2A presents TRIR spectra of 2 ML CdSe/CdS core/shell QDs arrayed in a solid film following optical excitation at 532 nm and 20 μJ/cm^2^ intensity per excitation pulse. As before, the film thickness, optical absorption at 532 nm (Figure S1B), and excitation intensity were chosen to minimize the probability that one quantum dot would absorb two photons^59^ per excitation pulse. The TRIR spectra again represented averages over time ranges that varied according to the time delay as listed in Table S1. The transient infrared absorption spectra exhibited markedly different broad electronic absorption offsets in comparison to the CdSe core-only QD measurements described above (Figure 1). This was because the CdS shell allowed electrons to delocalize over the entire nanostructure,^47^ which reduced their quantum confinement. This caused the 1S_e_-1P_e_ energy gap to decrease, resulting in a red-shift of the broad 1S-1P transition of electrons into the frequency range where ligand carboxylate features appear.

**Figure 2:**
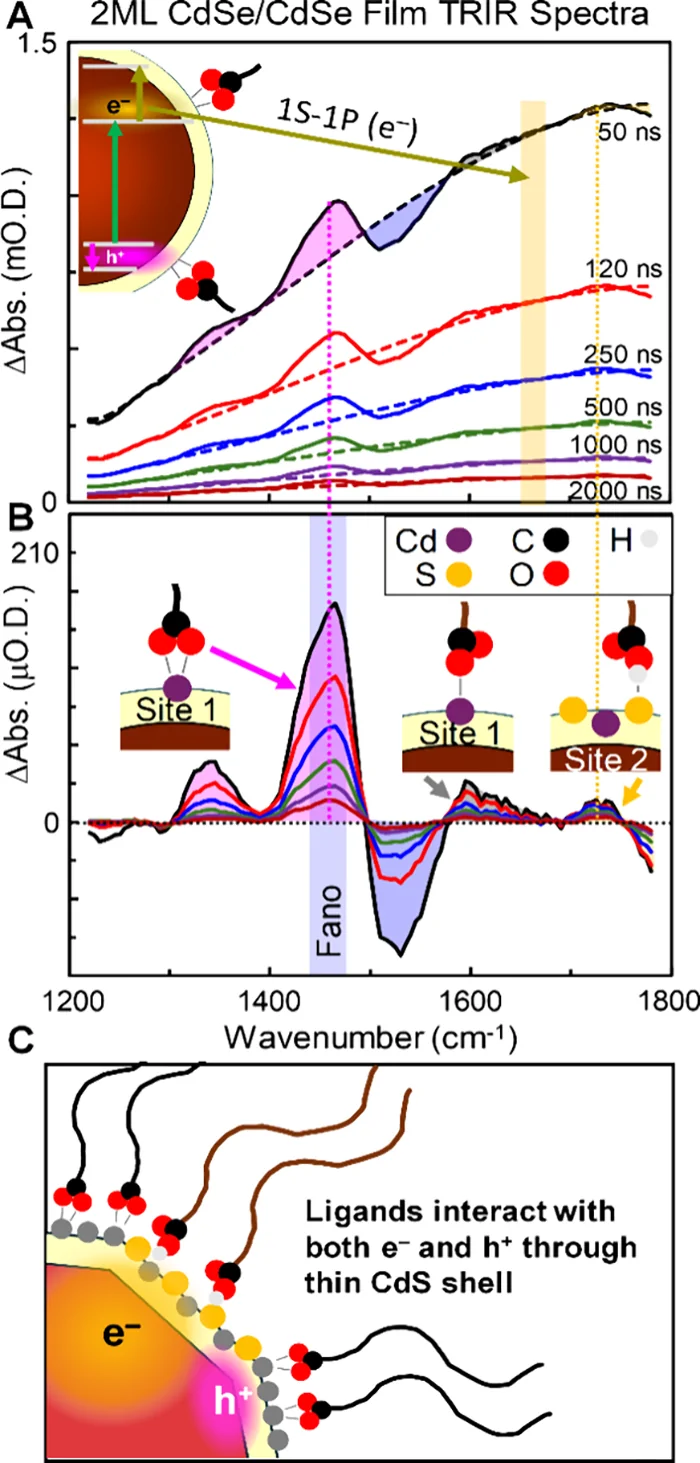
(**A**) TRIR spectra of a 2 ML CdSe/CdS QD film measured following 532 nm excitation. The transient vibrational features of the ligands are again superimposed on broad electronic absorption offsets (dotted lines). The yellow vertical box marks where the kinetics of the broad electronic absorption were measured. (**B**) Transient vibrational spectra obtained by subtraction of the broad electronic offsets from the measured TRIR spectra at each time delay. The pink and blue shaded peaks again highlight ‘hole associated’ carboxylate Fano-type vibrational features of the ligands as similarly highlighted in panel A. The vertical blue shaded box marks frequencies where the kinetics of the Fano-type resonance were measured after subtraction of the electronic offsets. (**C**) Cartoon illustration of the 2 ML CdSe/CdS QD surface highlighting the interactions of the carboxylate ligand vibrations with electron and hole density in the excited states of the QDs.

To isolate the transient vibrational features of the ligands attached to the 2 ML CdSe/CdS core/shell QDs, we fit the electronic absorption offsets with third-order polynomial functions (dotted curves in Figure 2A) and subtracted them from each TRIR spectrum. The transient vibrational spectra resulting from this subtraction appear in Figure 2B using the same color scheme for each time delay. Pink and blue shading were used to highlight the transient vibrational features in both Figure 2A and 2B for clarity. These features closely resembled the hole-associated transient vibrational features measured of CdSe core-only QDs appearing in Figure 1C despite the 2 ML CdSe/CdS QDs being conformally coated by a thin CdS shell. This finding revealed that the 2 ML CdS shell was thin enough to allow the surface bonded carboxylate groups to retain coupling to hole density localized in the CdSe cores. As we will show in the 6 ML CdSe/CdS QDs, the thicker CdS shells suppressed this coupling and prevented such hole-associated carboxylate features from being observed in the transient vibrational spectra of those samples.

The transient vibrational spectra of the 2 ML CdSe/CdS core/shell QDs in Figure 2B exhibited small features in the 1600 and 1700 cm^–1^ ranges that were higher in frequency than the hole-associated carboxylate features of their surfaced bonded ligands. These higher frequency features have been assigned to formal double bonded C=O moieties associated with monodentate (1600 cm^–1^) and free acid (1700 cm^–1^) carboxylate groups on the basis of comparisons of X-ray crystallographic data with infrared absorption spectra of a series of carboxylate metal salts and coordination states.^54,55^ The appearance of these transient vibrational features indicated weakening or photodetachment of ligands from the QD surfaces. Our recent ligand density dependent studies^46^ revealed that such photodetachment processes are driven by interactions of ligands with surface electron density in the excited states of the QDs. Therefore, the data revealed that the thin CdS shells of the 2 ML CdSe/CdS QDs represent an intermediate structure where a fraction of surface bonded ligands could still interact with hole density in the CdSe cores while other ligands could interact with surface electron density that delocalized through the shells. Figure 2C depicts a cartoon illustrating the variety of interactions ligands could have with surface electron and hole density in the excited states of the QDs. We note that because the 1600 and 1700 cm^–1^ transient vibrational features were likely Fano-type resonances, which gain oscillator strength by coupling to the 1S-1P electronic transitions of the QDs, it was challenging to interpret the amplitudes of the transient absorption features in terms of the corresponding changes in concentration of the perturbed or photodetached ligands.

We used TRIR measurements of the 6 ML CdSe/CdS core/shell QDs represented in Figure 3A to investigate the influence that shell thickness had on their excited state surface chemistry and ligand photodetachment dynamics. We hypothesized that the thicker CdS shell would insulate surface bonded carboxylate groups from hole density in the CdSe cores, leading to changes in the excited state surface chemistry of the nanocrystals. The TRIR spectra were measured of 6 ML CdSe/CdS QDs arrayed in a solid film following optical excitation at 532 nm and 20 μJ/cm^2^ intensity per excitation pulse. This excitation intensity, film thickness, and optical absorption at 532 nm (Figure S1C) again minimized the probability that one quantum dot would absorb two photons per excitation pulse. The TRIR spectra exhibited the prominent 1S-1P intraband transition of electrons that was further red-shifted relative to the 2 ML CdSe/CdS core/shell sample because of the increased delocalization of electrons in the thicker shell.

**Figure 3:**
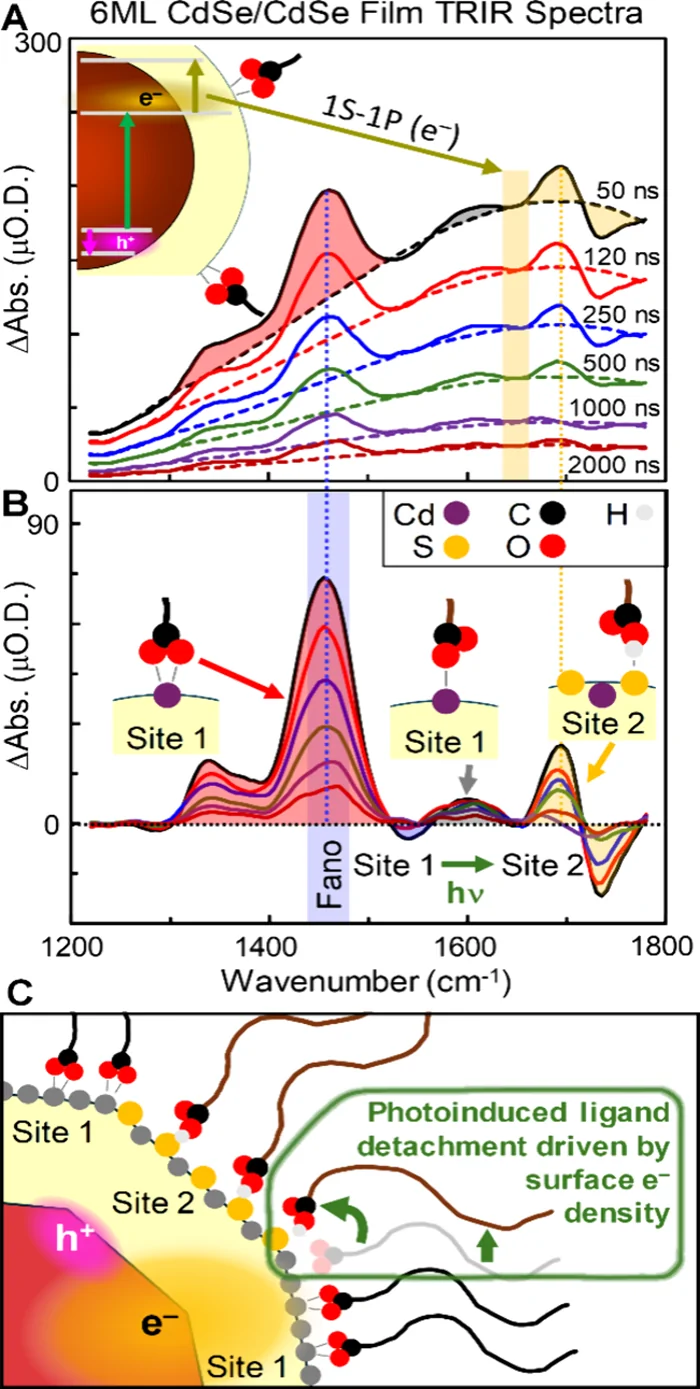
(**A**) TRIR spectra of a 6 ML CdSe/CdS QD film measured following 532 nm excitation. The ligand transient vibrational features and broad electronic absorptions were superimposed as in the previous samples. The yellow vertical box marks where the kinetics of the broad electronic absorption were measured. (**B**) The transient carboxylate vibrational features obtained by subtraction of the broad electronic offsets are represented at the same range of time scales. The carboxylate features in the 1300–1550 cm^–1^ region appear as all-positive Fano-type resonances. Importantly, the new transient vibrational features around 1700 cm^–1^ indicate that carboxylate ligands were photodetached from the QD surfaces, became protonated, but remained close enough to the surfaces to remain coupled to the surface electron density. (**C**) Cartoon illustration of the 6 ML CdSe/CdS QD surface highlighting 1) the photodetachment of the ligands, 2) their protonation, and 3) their reattachment as weakly bound protonated ligands. The layout of the cartoon and identification of the Site 1 and Site 2 surfaces were adapted from ref 50.

Following our established practices, the broad 1S-1P electronic transitions in each spectrum were fit with third-order polynomial functions (dotted lines in Figure 3A) and subtracted from the TRIR spectra to obtain the transient vibrational spectra appearing in Figure 3B. The spectra are plotted with the same color scheme corresponding to the time delays and time ranges as indicated in Table S1 as were used in Figures 1 and 2. The transient vibrational features of carboxylate groups of ligands bonded to the nanocrystal surfaces in the 1300–1500 cm^–1^ region appeared with all-positive spectral shapes, which contrasts with the QD samples with no or thin CdS shells. Red shading was used to highlight these all-positive transient vibrational features in the original data (Figure 3A) and in the results of subtraction of the electronic baseline (Figure 3B).

We showed in our recent ligand-density-dependent studies^46^ that such all-positive Fano-type resonances arose from carboxylate groups interacting with surface electron density in the nanocrystals. This observation confirmed that the thicker CdS shells of the 6 ML CdSe/CdS QDs insulated the surface bonded carboxylate groups from hole density in the CdSe cores. The appearance of a small transient vibrational feature at 1600 cm^–1^ was consistent with this surface electron density causing a fraction of the ligands to weaken their bonding by formation of monodentate structures as illustrated by the cartoons in the inset.

More importantly, the vibrational features around 1700 cm^–1^ in the transient vibrational spectra in Figure 3A and 3B (gold colored shading) indicated the formation of protonated carboxylate groups of ligands that photodetached from the 6 ML CdSe/CdS QDs.^46^ For completeness, we showed in Figure S4 an FTIR spectrum of oleic acid dissolved in CCl_4_ to confirm that free carboxylic acid groups had unique absorption peaks at 1700 cm^–1^ that were distinct from the frequencies of carboxylates that are deprotonated and bonded to the QD surfaces.^54,55^ The assignment of the 1700 cm^–1^ vibrational feature to photodetached and protonated ligands was further supported by two observations from our previous work in ref 46. First, we found that the density of ligands on CdSe QD surfaces influenced their surface electron trap density as revealed by changes of their excited state lifetimes. Second, we found that the QD surfaces with more electron traps exhibited more photoinduced ligand detachment, at least of X-type ligands such as carboxylates. Applying these findings to the 6 ML CdSe/CdS core/shell QD samples, we concluded that the larger amplitude of the Fano-type vibrational feature at 1700 cm^–1^ in Figure 3B, which we recall is unique to carboxylic acid groups that are protonated (Figure S4), confirms that the thicker shells of the 6 ML CdSe/CdS QD samples localized surface electron density as expected and that this behavior enhanced the fraction of ligands that photodetached from the QD surfaces and became protonated.

Importantly, the vibrational features around 1700 cm^–1^ still exhibited a derivative line shape that was characteristic of Fano-type resonances.^56,57^ The FTIR spectrum of the 6 ML CdSe/CdS QD sample used for these TRIR measurements appears in Figure S3 and indicated that only a small fraction of ligands in the sample existed as free acids at equilibrium. Therefore, the appearance of the 1700 cm^–1^ Fano-type resonance confirms that carboxylate ligands underwent photoinduced detachment and then became protonated. However, because the sample consisted of a dense solid film, the photodetached and protonated ligands were unable to diffuse far from the nanocrystal surfaces. This caused the ligands to remain close enough to their surfaces to be coupled to the excited electronic states of the QDs. Comparison of FTIR spectra in Figure S3 of the 6 ML CdSe/CdS QD film before versus after prolonged exposure to the laser excitation pulses revealed that photodetached ligands were able to deprotonate and reattach to the nanocrystal surfaces between excitation pulses.^18,46^

We sought a test of the validity of the assignments described above for the types of charge carriers (electrons or holes) that were responsible for the broad electronic absorptions and Fano-type ligand vibrational features of the various QD samples. We established this validity by comparing the decay kinetics of the 1S-1P electronic absorption offsets in each sample with the corresponding decay kinetics of their Fano-type resonances. We reasoned that if both types of spectral features arose from the same charge carrier in a given sample, then the decay kinetics of the electronic and vibrational features should match quantitatively. We first compared in Figure S5 the excited state decay kinetics measured from the 1S-1P broad electronic absorption offsets in each of the three QD samples. The pink shaded box in Figure 1B and the yellow shaded vertical boxes in Figures 2A and 3A indicate the frequencies where the electronic absorption kinetics were measured in each sample. Triexponential fit functions were used to quantify the decay traces and are overlaid on the transient absorption decay kinetics in the figure. The best fit parameters and uncertainty limits from the fitting procedure are tabulated in Table S2. The amplitude-weighted average time constants of the kinetic decays obtained from this procedure were 55 ± 10 ns, 300 ± 30 ns, and 530 ± 70 ns for the CdSe core-only, 2 ML CdSe/CdS, and 6 ML CdSe/CdS QD samples, respectively. The uncertainty limits of these average time constants were dominated by the uncertainty of the amplitudes of the longest decay components of the kinetics of each sample. As expected, the data revealed that the 6 ML CdSe/CdS sample had the longest excited state lifetime, which was consistent with prior observations.^45^

We note that the increase of the excited state lifetime of the 6 ML CdSe/CdS core/shell QD sample excludes an alternate explanation of the enhanced photoinduced ligand detachment observed in that sample. We observed in our recent work^46^ that CdSe QDs with lower ligand shell densities exhibited greater extent of photoinduced ligand detachment. Therefore, an alternate argument could be suggested that the greater extent of ligand photodetachment observed in the 6 ML CdSe/CdS sample could be a result of a lower ligand shell density. However, a lower ligand shell density would also be expected to increase the surface electron trap density, which should decrease the excited state lifetime. The opposite is observed (Figure S5), which eliminates the lower ligand density as a explanation for the enhanced photodetachment observed in the 6 ML sample.

Figure 4 compares the decay kinetics of the broad absorption offsets with the time dependence of the Fano-type vibrational features measured in each sample. The kinetics decay trace labeled ‘Fano (h^+^)’ in Figure 4A indicated the time dependence of the hole-associated carboxylate vibrational features obtained from the amplitude of the vibrational feature between 1440 and 1470 cm^–1^ (blue vertical shaded band in Figure 1C) after subtraction of the 1S-1P transition of holes. The kinetics trace labeled ‘1S-1P (h^+^)’ was reproduced from Figure S5 and reflected the time dependence of the hole population. The comparison revealed that both the Fano-type hole-associated carboxylate features and the 1S-1P transition of holes decayed on the same time scale. The best triexponential fits of the kinetics traces revealed average lifetimes of 70 ± 15 ns and 55 ± 10 ns, which differ but were not statistically distinguishable within experimental precision. The best fit parameters of the Fano (h^+^) kinetics are tabulated in Table S3. The comparison confirmed that both the vibrational and electronic transitions decayed synchronously because they arose from interactions with hole density in the excited states of the CdSe core-only QDs.

**Figure 4:**
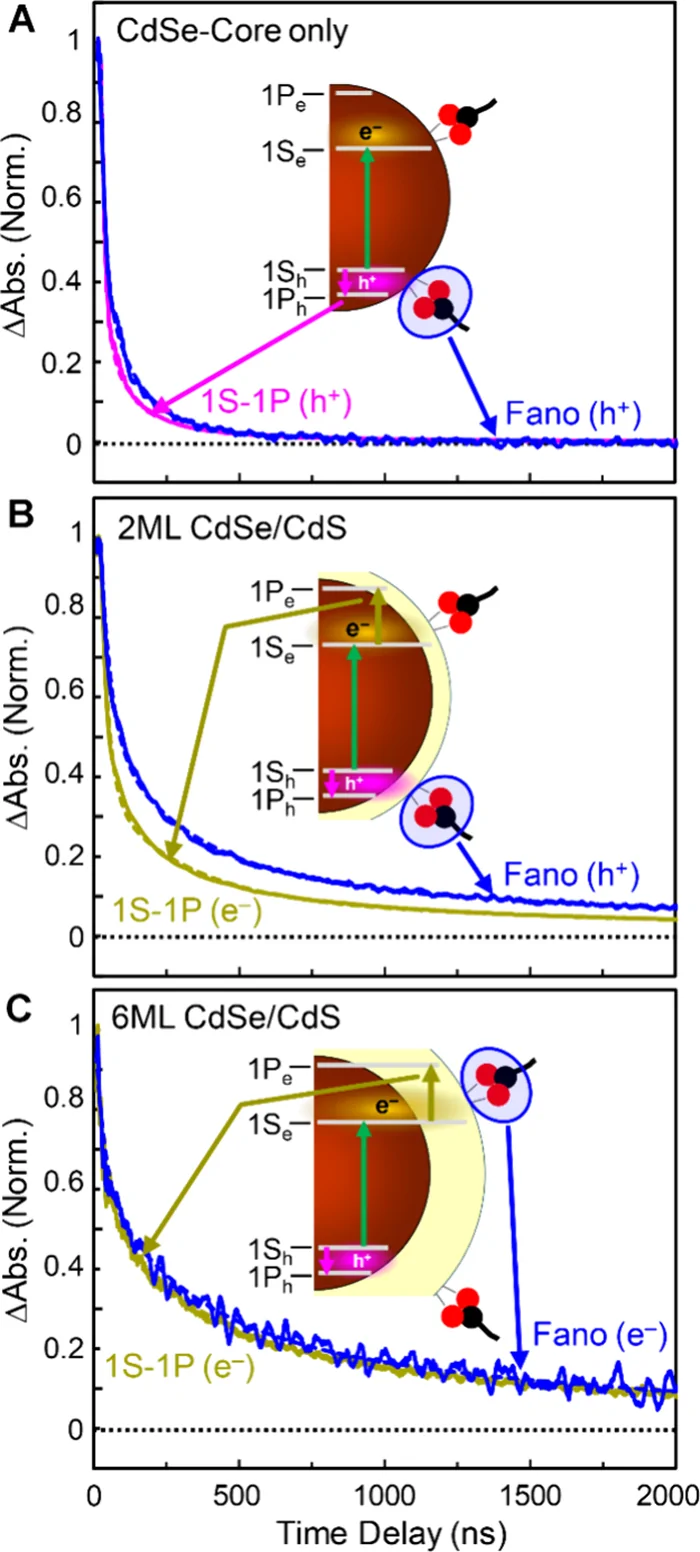
Comparison of decay kinetics of the broad 1S-1P electronic transitions versus the Fano-type vibrational transitions measured in (**A**) CdSe core-only, (**B**) 2 ML CdSe/CdS, and (**C**) 6 ML CdSe/CdS QD films. The decay kinetics of the Fano-type and 1S-1P transitions in the CdSe core-only and the 6 ML CdSe/CdS QDs match each other because both arise from the same type of charge carrier in their respective nanocrystals. However, the thinner shells in the 2 ML CdSe/CdS QDs allow ligands to interact with holes in the CdSe cores, while the 1S-1P transition results from electrons. This causes their kinetics to differ in these QDs.

However, we expected that the Fano-type hole-associated carboxylate features in the 2 ML CdSe/CdS core/shell QDs would not match the kinetic decay of the 1S-1P transition of electrons in that sample. This was because the transitions arose from interactions with different types of charge carriers. The time dependence of the 1S-1P transition of electrons in the 2 ML CdSe/CdS reflected the population of electrons in the delocalized states of the QDs, which could decay by electron–hole recombination and also by localizing into charge traps at the nanocrystal surfaces. In contrast, because the CdS shells passivated dangling bonds at the surfaces of the CdSe cores, the decay of hole density should be dominated by electron–hole recombination alone. Similar to our procedure for the CdSe core-only QDs, the kinetic decay trace labeled ‘Fano (h^+^)’ in Figure 4B indicated the time dependence of the hole-associated carboxylate vibrational features (blue vertical shaded band in Figure 2B) after subtraction of the broad electronic absorption offsets. The kinetics trace labeled ‘1S-1P (e^–^)’ was reproduced from Figure S5 and reflected the time dependence of electrons in the delocalized states of the 2 ML CdSe/CdS QDs. The comparison revealed that the Fano-type hole-associated carboxylate features indeed decayed more slowly than the 1S-1P transition of electrons. The best triexponential fit of the Fano (h^+^) kinetics trace revealed an average lifetime of 460 ± 50 ns in comparison to the 300 ± 30 ns average lifetime of the 1S-1P intraband transition. This difference in average lifetimes of the Fano features versus the electronic transitions confirmed that they reflect the population decay of different charge carriers.

Finally, as discussed above, the change in shape of the Fano-type vibrational features in the 6 ML CdSe/CdS core/shell QD sample (Figure 3B) indicated that they arose from interaction of carboxylate groups with surface electron density.^46^ This reasoning therefore led us to expect that the time dependence of the Fano-type features of the 6 ML CdSe/CdS core/shell QD sample should again match the kinetic decay of the broad electronic transition because both types of transitions arose from interactions with surface electron density. Figure 4C presents the kinetic decay labeled ‘Fano (e^–^)’, which was obtained from the amplitudes of the Fano-type features of the sample measured between 1440 and 1470 cm^–1^ after subtraction of the broad electronic absorptions (see blue vertical shading in Figure 3B). The kinetics trace labeled ‘1S-1P (e^–^)’ was reproduced from Figure S5. The best triexponential fits of the kinetics traces indicated average lifetimes of Fano and 1S-1P kinetics of 570 ± 70 ns and 530 ± 70 ns (see Tables S2 and S3), which were again indistinguishable within experimental precision. In total, the analysis of the kinetic decays in Figure 4 confirmed our assignments of the types of charge carriers that gave rise to the 1S-1P and Fano-type vibrational transitions in each sample.

Having confirmed our assignments, it was useful to compare the photoinduced ligand detachment processes that we observed here with insights from recent NMR studies of the distributions of surface bonded carboxylate ligands on QDs.^50–52^ In particular, the vinyl protons of oleic acid ligands were used to identify distinct carboxylate bonding environments on the surfaces of colloidal QDs. One population of ligands were more strongly bonded to the nanocrystal surfaces and were in their deprotonated state. These ligands were associated with ‘Site 1’ regions of the QD surfaces as illustrated in Figures 2B and 3C.^50^ These sites were identified as metal-rich [100] facets. Another population of ligands was identified that were protonated but still bonded to the nanocrystal surfaces. These ligands were more weakly bonded and were associated with ‘Site 2’ regions of the nanocrystal surfaces, which were assigned to [111] facets.^50^ The partitioning of ligands among these surface bonding environments and protonation states was found to occur under equilibrium conditions and to be dependent on the concentration of ligands in solution and their surface density on the QDs.

Combining observations from the NMR studies with our TRIR measurements of the 6 ML CdSe/CdS core/shell QDs revealed that it was the enhancement of surface electron density in the CdS shells that led to photoinduced ligand rearrangements of the type illustrated in Figure 3C. In particular, deprotonated ligands initially bonded to nanocrystal surfaces at Site 1 regions underwent photoinduced ligand detachment and remained detached long enough to become protonated. These protons were likely donated from ligands already present in the weakly bonded Site 2 geometries.^50^ However, because the photodetached and protonated ligands could not diffuse far from the QD surfaces, they remained close to the nanocrystals and retained their coupling to the surface electron density, leading to the Fano-type resonance of the free acid peak around 1700 cm^–1^. We speculate that this type of photoinduced ligand detachment driven by surface electron density may be unique to X-type ligands such as carboxylates or phosphonates. It will be interesting to determine in future work whether such photodetachment dynamics are observed in other ligand types such as thiols on nanocrystal surfaces.

The similarity in amplitude of the Fano-type vibrational features around 1450 cm^–1^ and those of the photodetached and protonated ligands around 1700 cm^–1^ in the 6 ML CdSe/CdS QDs (Figure 3B) suggested that a significant fraction of the ligands that were perturbed in the excited states of the nanocrystals ended up photodetaching and becoming protonated. We recognize that it was challenging to quantitatively interpret the amplitudes of the Fano-type vibrational features because their enhancement factors arising from their coupling to the 1S-1P electronic transition were unknown. Nonetheless, the similarity in amplitude of these vibrational features was intriguing. We recently showed that photoinduced ligand detachment occurred with near unit quantum yields from CdSe core-only QDs in solution.^46^ Our findings here suggested that an even greater number of ligands may be perturbed per absorbed photon by surface electron density in the excited states of the 6 ML core/shell QDs.

We note that the longer excited state lifetime of the 6 ML CdSe/CdS QDs (Figure S5) caused excited state surface electron density to persist for longer periods of time in the thicker-shell sample in comparison to the others. This persistent excited state surface electron density may also have contributed to the greater extent of photoinduced ligand detachment and protonation observed in Figure 3B. This observation suggested a design principle that we plan to explore in forthcoming work. Namely, colloidal QDs with longer excited state lifetimes caused by segregation of electron density to their surfaces may enable greater transient reduction of the density of their ligand shells during photocatalytic processes. The reversibility of this process in solution^46^ suggested that it may be possible to use this approach to enhance the photocatalytic activity of colloidal quantum dots by modulating the porosity of their ligand shells while allowing restoration of those shells between excitation events.

In summary, we investigated the influence that nanocrystal architecture had on photoinduced ligand detachment from colloidal QDs in solid films following optical excitation to their excitonic states. We examined a series of CdSe core-only and CdSe/CdS core/shell QDs with different shell thicknesses and excited them using photons tuned to their excitonic bandgaps. We monitored using time-resolved infrared spectroscopy the extent of photoinduced ligand detachment as a function of QD architecture. We found that CdSe core-only QDs (no shells) exhibited hole-associated Fano-type transient vibrational features that resulted from coupling of ligand carboxylate anchoring groups to surface localized hole density. However, these QDs exhibited little photoinduced ligand detachment when cast in solid films. Similarly, CdSe/CdS core/shell QDs with shells thin enough to still allow ligand carboxylate anchoring groups to interact with hole density in their cores also did not exhibit significant photoinduced ligand detachment in solid films. However, CdSe/CdS core/shell QDs with thicker CdS shells exhibited substantial photoinduced detachment and protonation of their ligands as indicated by Fano-type vibrational features at 1700 cm^–1^. Because the film environment restricted the diffusive motion of these photodetached and protonated ligands, they were forced to remain close to the nanocrystals and so retained their coupling to the electronic states of the nanocrystals. Our findings suggested that nanocrystal architectures that segregate electron density at their surfaces and sustain that density for longer periods of time were likely to exhibit photoinduced ligand detachment to a greater extent. In future work, we will explore this design principle in other nanocrystal architectures that may exhibit more extensive photoinduced ligand detachment.

## Supplementary Material



## Notes

Detailed experimental procedures, absorption and PL spectra of CdSe/CdS core/shell QDs, time averaging methods for TRIR spectra, PL quenching measurements to confirm core/shell architecture, FTIR spectra of surface-bonded ligands before versus after prolonged laser exposure, FTIR spectra of free oleic acid molecules in solution, detailed kinetic analysis and comparison of excited state decay kinetics of core/shell QDs (PDF)
