# Supplementary material for: Control of Photoinduced Ligand Detachment from Quantum Dot Surfaces Using Nanocrystal Architecture
Source: J Phys Chem Lett. 2026 Aug 10;17(33):9660–8. doi: 10.1021/acs.jpclett.6c01698 (PMC13501947; doi:10.1021/acs.jpclett.6c01698)
Supplement: Supplementary file 1 [file jz6c01698_si_001.pdf]

Supporting Information for:

Control of Photoinduced Ligand Detachment from Quantum Dot Surfaces Using Nanocrystal Architecture

McKenna N. Grega, Jacob A. Cho, Robert A. Brown, and John B. Asbury*

Department of Chemistry, The Pennsylvania State University, University Park, PA 16802, USA.

Table of Contents:

Section S1: Experimental Methods.

Section S2: Comparison of absorption and PL spectra of CdSe core-only and CdSe/CdS core/shell QDs with varying shell thicknesses.

Section S3: Time averaging method to produce TRIR spectra.

Section S4: PL quenching experiments using thiols to verify the core/shell architecture of CdSe/CdS QDs.

Section S5: FTIR spectra of the QD samples measured before versus after prolonged photoexcitation during the TRIR measurements.

Section S6: FTIR spectrum of OA in CCl_4 showing free carboxylic acid absorption

Section S7: Comparison of the excited state decay kinetics measured from electron and hole 1S-1P intraband transitions of the QDs.

Section S8: Literature Cited

Section S1: Experimental Methods

CdSe QD SYNTHESIS: The synthesis of CdSe QDs was adapted from a procedure outlined by Liulin and Jiongzhao et al.[1] For ~2.5 nm QDs, 0.0256g CdO, 0.1580g stearic acid and 4.0 mL 1-octadecane (ODE) were heated to 120 ℃ under vacuum to degas the precursors. Once the temperature reached 120 ℃, the reaction flask was kept under N_2 flow for the remainder of the nucleation process. While the reaction flask was heated, 0.0237g elemental Se was added to 3.0 mL ODE and 600 uL oleylamine and sonicated for ten minutes until a Se suspension formed. The reaction flask was heated to 250 ℃ causing the solution to become clear. Then, 1.0 mL of the Se-ODE suspension was injected. The reaction flask was then kept at 250 ℃ for 15 minutes. Following the 15- minute reaction time, the reaction flask was removed from heat and allowed to cool to 200 ℃ before using a water bath to cool the flask to room temperature. A 50 ℃ water bath was prepared, containing three test tubes each with 3.0 mL of a 1:1:1 mixture of methanol: acetone: chloroform. Once the reaction flask cooled, the orange product was evenly distributed amongst the three test tubes and heated in the 50℃ water bath until no layers persisted. The three test tubes were then subjected to centrifugation at 4000 RPM for 30 seconds, and the supernatant containing excess and stearate and ODE was discarded. The precipitate was washed once more using the same procedure, and the collected precipitate was capped in a test tube and allowed to dry overnight.

CdSe/CdS QD SYNTHESIS**:** The synthesis of CdSe/CdS QDs was adapted from a procedure outlined by Cirillo et al.[2] For 2 ML CdSe/CdS QDs, 0.0078g CdO and 200 uL oleic acid (OA), (6 ML: 0.024g CdO and 400 uL OA) were added to 3.0 mL ODE and heated to 120 ℃ under vacuum to degas the precursors. Once the reaction temperature reached 120 ℃, the flask was kept under N_2 flow for the remainder of the reaction process. Once the reaction flask reached 250 ℃ and the solution color turned clear, 1.8 mL trioctylphosphine (TOP) was injected. Once the reaction flask temperature recovered to 250 ℃ after the addition of TOP, a solution containing 1.8 mL TOP, 1.0 mL OA, 0.005g CdSe, and 0.015g elemental sulfur was injected, and the reaction flask was kept at 250 ℃ for three minutes. Following the three- minute reaction time, a water bath was used to cool the reaction flask to room temperature. 5.0 mL toluene was added to the crude solution before washing with a 1:1:1 ratio of isopropanol: ethanol: crude product in three test tubes. The test tubes were then subjected to centrifugation at 4000 RPM for 10 minutes, and the supernatant containing the excess ligand and solvent was discarded. This washing procedure was repeated once more before the collected precipitate was redispersed in toluene.

SAMPLE PREPARATION: CdSe/CdS films were prepared by first depositing ~100 nm thick mesoporous alumina adhesion layers onto CaF_2 substrates. The adhesion layers were prepared using 1.0 mL of a commercial 20% weight alumina-nanoparticle suspension that was diluted with 3.0 mL of isopropanol followed by sintering. After the substrates cooled completely, the nanocrystal films were deposited by drop casting 300 uL of a CdSe/CdS-toluene solution onto the alumina adhesion layers and allowed to dry completely.

TRANSIENT ABSORPTION: Mid-IR transient absorption experiments were performed using a table top transient absorption instrument, which consisted of a pulsed, frequency doubled (532 nm) Nd:YAG laser operating at 5 kHz as the excitation source and a SiC globar as the probe light source. The probe light source was dispersed in a monochromator and detected with a cryogenically cooled mercury cadmium telluride (MCT) photovoltaic detector. All transient absorption measurements were conducted inside a cryostat under dry N_2 to avoid exposure to oxygen during the measurements.

PHOTOLUMINESCENCE: Steady-state and time-resolved PL measurements were conducted on a visible transient absorption system with the probe beam blocked. The system consisted of a pulsed, frequency doubled (532 nm) Nd:YAG laser operating at 10 kHz or a frequency tripled (355 nm) Nd:YAG laser operating at 30 Hz as the excitation source. The photoluminescence from the sample was dispersed in a monochromator and detected using photodiodes optimized for detection in the visible. The time-dependent response of the photodiode detector was integrated to produce the steady-state PL spectra.

STEADY STATE OPTICAL MEASUREMENTS: Visible absorption measurements were performed with a Beckman DU 720 UV/Vis spectrometer. The spectra were background subtracted with a blank CaF_2 substrate. FTIR spectra were measured using a commercially available instrument (JASCO, 6600 FTIR Spectrometer equipped with a liquid nitrogen cooled MCT detector).

<u>Section S2</u>: Visible absorption and photoluminescence spectra of CdSe core-only and CdSe/CdS core/shell QDs with varying shell thicknesses.

Figure S1 illustrates the absorbance (solid lines) and emission spectra (dashed lines) of CdSe/CdS QDs examined in this work with varying shell thicknesses that all contained the same size CdSe cores. The systematic red-shift of the excitonic absorption and emission spectra of the QDs resulted from delocalization of electron density throughout the nanostructures, while the holes remained confined to the CdSe cores, as illustrated by the inset in each figure panel.

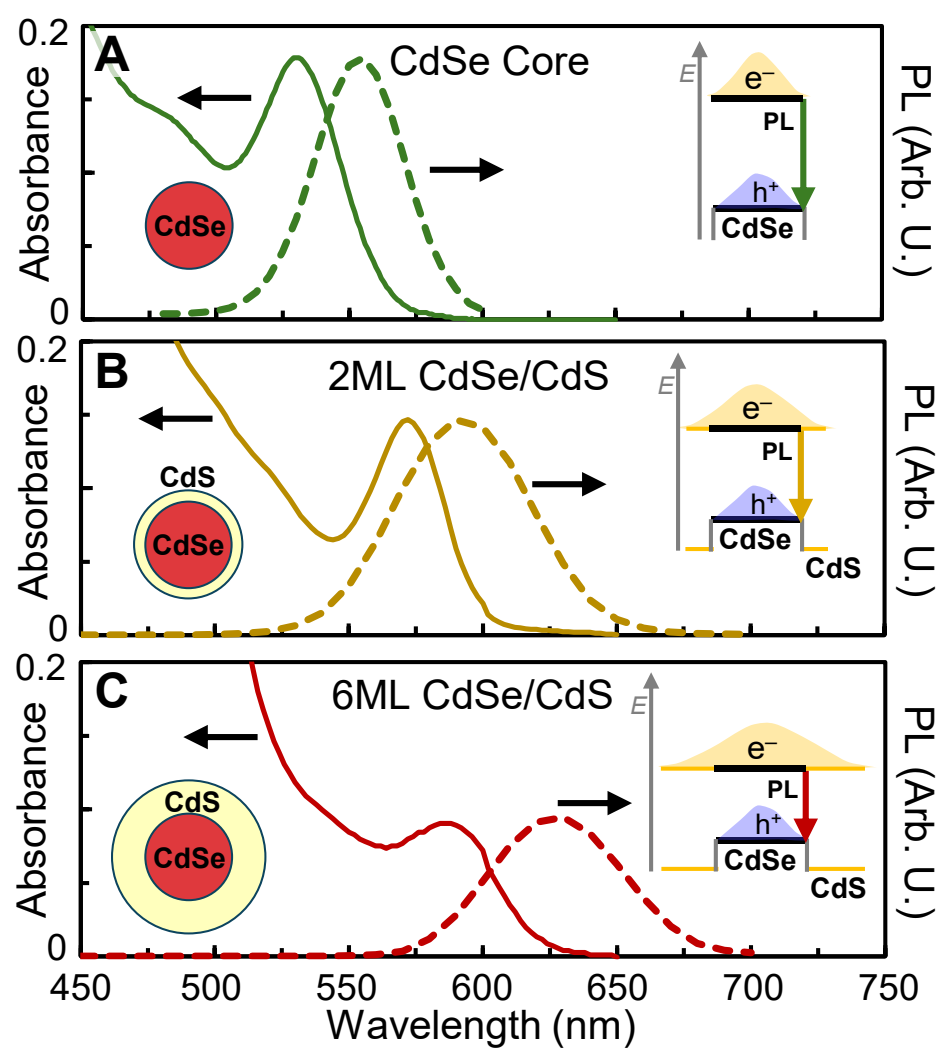

Figure S1. Absorbance and photo-luminescence (PL) spectra of CdSe core only (**A**.), 2ML CdSe/CdS core/shell (**B**.), and 6ML CdSe/CdS core/shell QDs (**C**.). The red-shift of the absorbance and PL spectra of QDs with thicker shells results from delocalization of electrons throughout the nanocrystals, while holes are confined to the CdSe cores.

Section S3: Time averaging method to produce TRIR spectra.

To enhance their signal-to-noise ratio, the TRIR spectra appearing in **Figures 1, 2, and 3** represent averages over multiple time points at each frequency. **Table S1** lists the full set of time ranges that were averaged to produce the TRIR spectra.

Table S1. List of the time ranges in nanoseconds over which the TRIR spectra were averaged to produce the data represented in this work.

Interval	Time Range	Interval	Time Range
1	50-70	2	120-150
3	250-300	4	500-600
5	1000-1400	6	2000-2400

<u>Section S4:</u> PL quenching experiments using thiols to verify the core/shell architecture of CdSe/CdS QDs.

Figure S2 represents the results of a PL quenching study used to verify that the synthesis of CdSe/CdS core/shell QDs produced conformal CdS shells around the CdSe cores. We added dodecanethiol to hexane solutions of the various QDs examined in this work because thiols are known to quench PL in CdSe QDs. The thiol groups bonded to QD surfaces introduce midgap states that trap holes, which splits excitons and therefore suppresses their radiative decay.[3-5] **Figure S2A** shows the results of adding 17 mM dodecanethiol to CdSe core-only QDs. The near complete suppression of PL from the CdSe QDs in the presence of thiols confirms their quenching behavior. In contrast, the addition of the same concentration of dodecanethiol thiol to hexane solutions of the 2ML and 6ML CdSe/CdS core/shell QDs did not result in quenching of their PL emission. The PL spectra in **Figure S2B** and **S2C** reveal nearly identical PL emission intensity before versus after the addition of thiols to both core/shell QD solutions. The slight red-shift of the PL spectra from the core/shell QDs in the presence of thiols is likely the result of the change of dielectric of the nanocrystal surfaces by exchange of their carboxylate ligands for the thiols. This red-shift confirms that the thiols did in fact attach to the core/shell QD surfaces but did not quench their radiative decay. We conclude therefore that the synthesis of both 2ML and 6ML CdSe/CdS core/shell QDs produced conformal CdS shells over the CdSe cores.

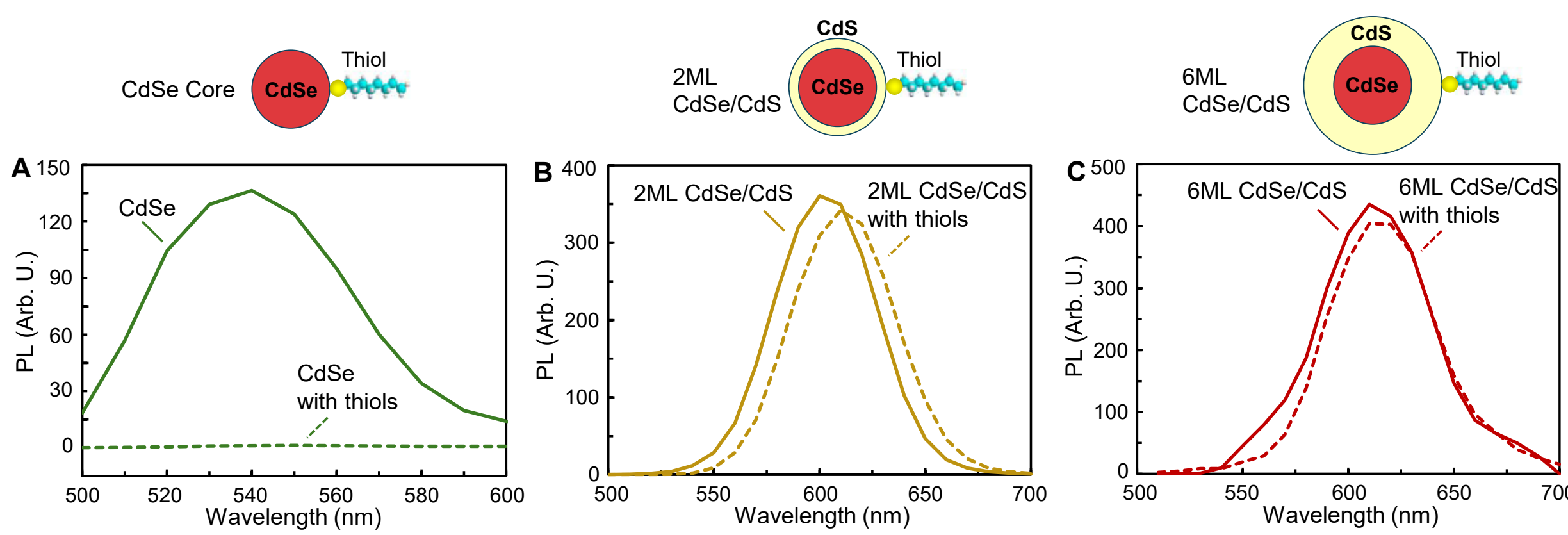

Figure S2. Comparison of PL spectra of QDs examined in this work with and without the presence of dodecanethiol in solution. (**A.**) The addition of thiols to solutions of CdSe core-only QDs leads to complete quenching of their excitonic emission. (**B.**) The growth of CdS shells around the CdSe cores of the 2ML CdSe/CdS QDs separates the mid-gap states formed by thiols on the CdS surfaces from hole density in the CdSe cores. This allows excitons to retain their radiative decay paths in the presence of thiols. (**C.**) The 6ML CdSe/CdS QDs also exhibit robust excitonic emission in the presence of thiols for the same reasons that the 2ML CdSe/CdS QDs retain their emission. This confirms the conformal coating of the CdSe cores by the CdS shells.

<u>Section S5</u>: FTIR spectra of the QD samples measured before versus after prolonged photoexcitation during the TRIR measurements.

Figure S3 depicts the results of a longevity study of the CdSe and CdSe/CdS core/shell QD films to explore the reversibility of the observed photoinduced ligand detachment process. The FTIR spectra were measured of the various QD films before versus after prolonged four-hour exposure to the 532 nm excitation laser used in the TRIR measurements. This excitation exposure led to every QD in the films undergoing ~10^7 excitation cycles on average. The data reveal negligible changes in the carboxylate absorption features of the surface-bound ligands. The small increase in absorption of the free acid peak around 1700 cm^{-1} in **Figure S3C** indicates that more than 99.99% of the photodetached and protonated ligands successfully restored their original bonding geometries within the 100 μs between excitation pulses during the experiment.[6]

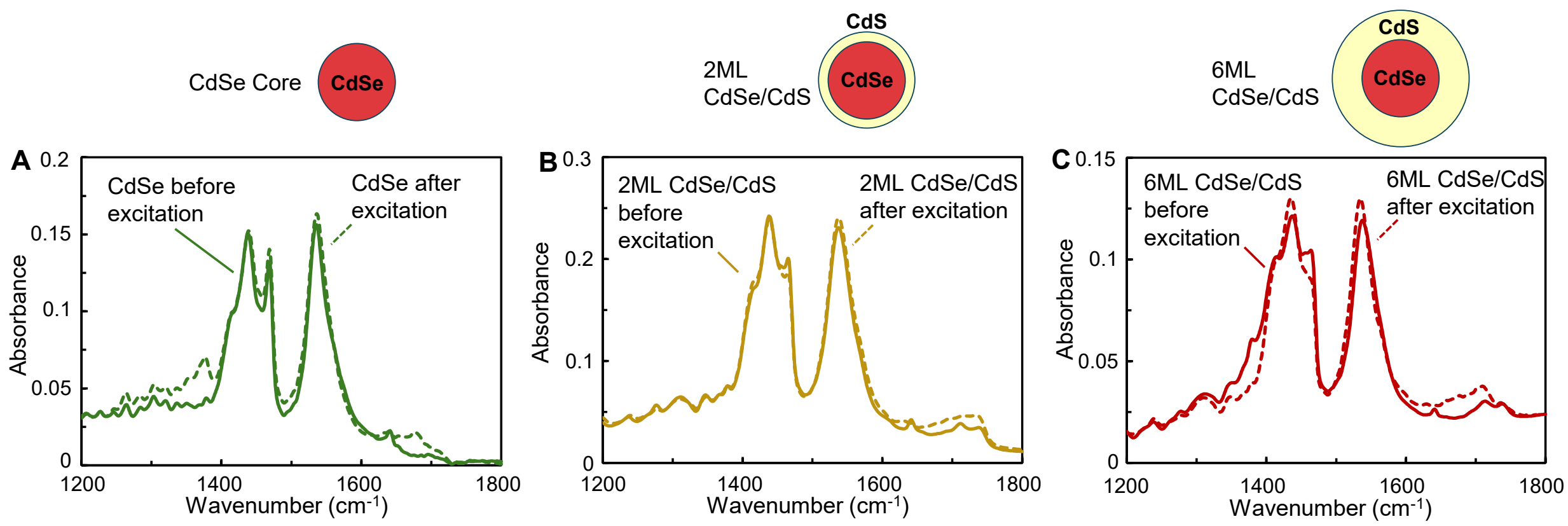

Figure S3. Comparison of FTIR spectra of the various CdSe (**A**.) and CdSe/CdS (**B**. and **C**.) QD films examined in this work before versus after prolonged excitation by the 532 nm optical pump pulse over the duration of the TRIR measurements.

Section S6: FTIR spectrum of oleic acid (OA) in CCl4 showing free carboxylic acid absorption

The FTIR spectrum of a 20 mM solution of neat oleic acid in CCl_4 measured with a 150 µm optical pathlength is represented in **Figure S4**. The free acid peak at 1720 cm^{-1} is apparent in the spectra and confirms that the 1720 cm^{-1} feature observed in the time-resolved infrared spectra of CdSe QDs in solution corresponds to the absorption of protonated oleic acid ligands. Importantly, the appearance of the free carboxylic acid absorption peak at this unique, higher frequency confirms that features observed around 1700 cm-1 in the TRIR spectra of the CdSe and CdSe/CdS QD samples correspond to protonated ligands. This confirms the assignment of the transient absorption peak around 1700 cm-1 in the 2ML and 6ML QD samples to ligands that photodetached from their surfaces, became protonated, but remained close enough to their surfaces to retain coupling to the electronic states of the nanocrystals.

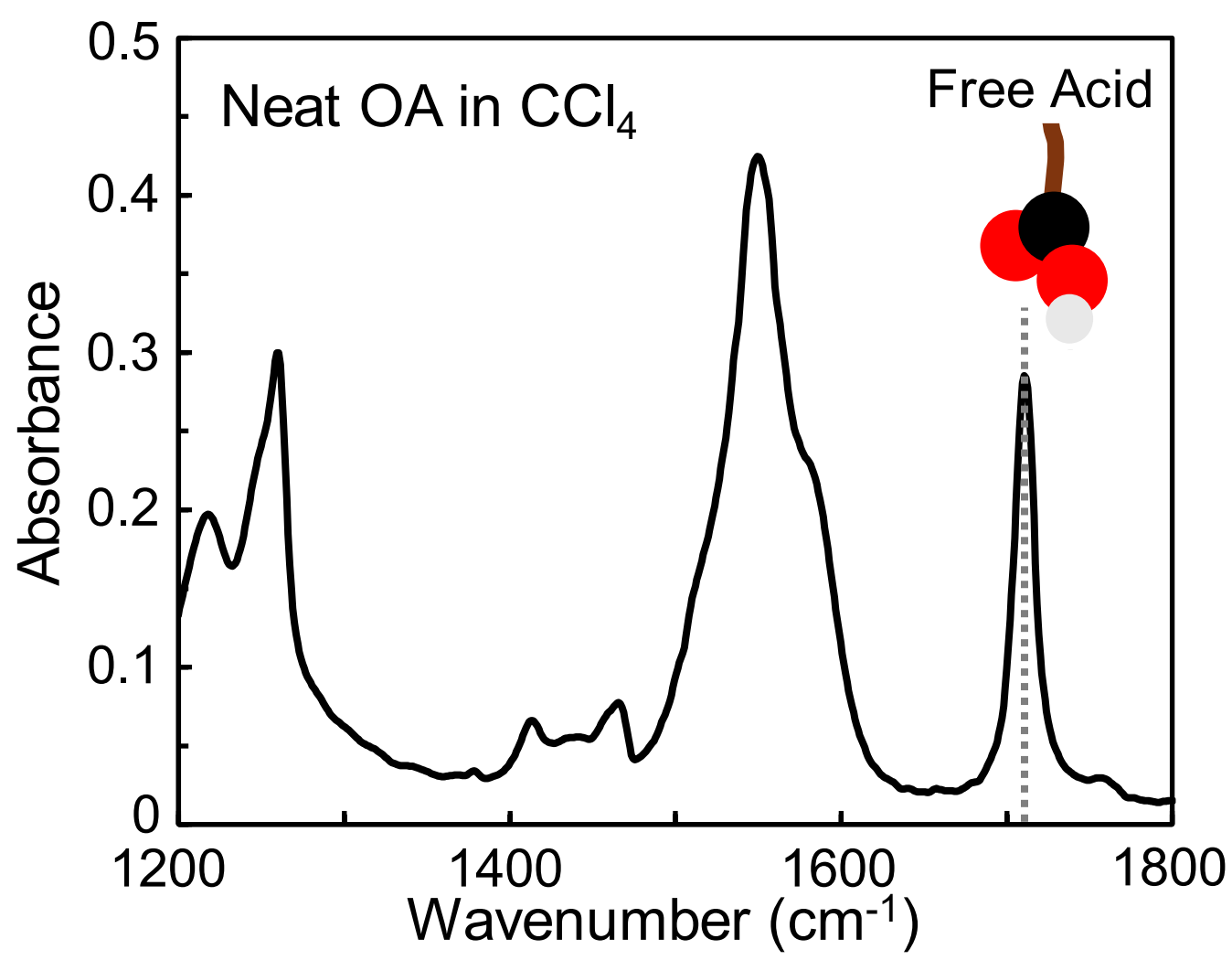

Figure S4. FTIR spectrum of oleic acid dissolved in CCl_4.

Section S7: Comparison of the excited state decay kinetics of the CdSe and CdSe/CdS core/shell QDs examined in this work.

We compared in **Figure S5** the effect that variation of the CdS shell thicknesses had on the time scales for excited relaxation of the QDs. The kinetics traces represent the time dependence of the amplitudes of the broad electronic transitions appearing in the TRIR spectra of the samples that were measured at frequencies where no vibrational features appeared. The pink shaded vertical band in **Figure 1B** indicates the frequencies where the kinetics for the CdSe core-only QDs were measured. This frequency corresponds to the 1S-1P intraband transition of holes in the QDs. The gold shaded vertical band in **Figures 2A** and **3A** indicate the frequencies where the kinetics for the 2 ML and 6 ML CdSe/CdS QDs were measured, respectively. These kinetics represent the time-dependence of the 1S-1P intraband transition of electrons in the QDs because the increased delocalization into the CdS shells caused this transition to red-shift into the spectral region of the carboxylate vibrational features. These assignments are illustrated by the inset with corresponding arrows connecting the kinetics with their associated QDs and CdS shell thicknesses.

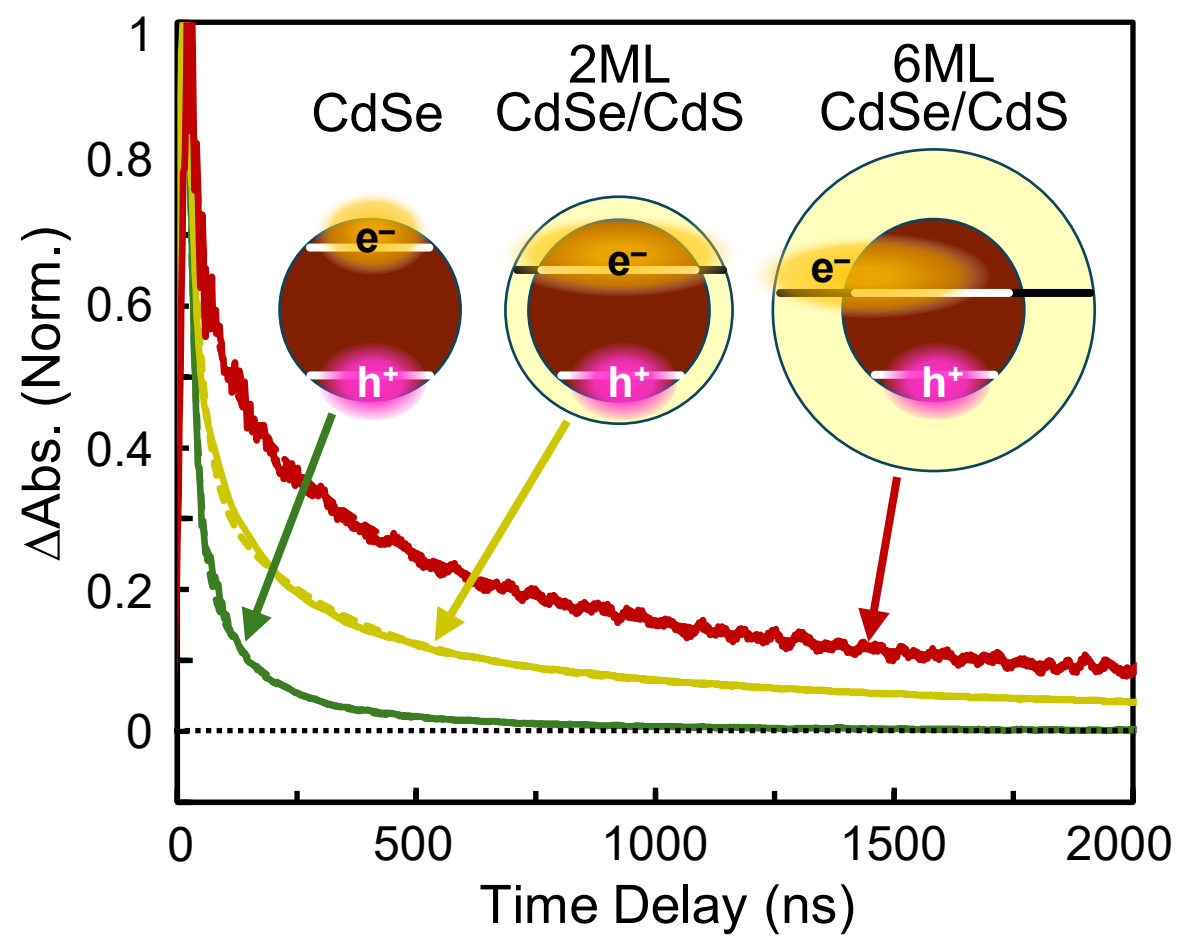

Figure S5. Decay kinetics of the broad electronic absorption offsets measured in CdSe core-only, 2ML CdSe/CdS, and 6ML CdSe/CdS QD films. The broad electronic transition of the CdSe core-only QDs corresponds to the 1S-1P intraband transition of holes and exhibits the shortest excited state lifetime. The broad electronic transitions of the 2ML and 6ML core/shell QDs exhibit markedly longer excited state lifetimes because the CdS shell provides superior passivation of midgap states at the CdSe core surfaces and because the presence of the CdS shell insulates the holes from nanocrystal surfaces. The longer excited state lifetime of the 6ML CdSe/CdS QDs is likely a result of greater insulation of holes in the CdSe cores from the nanocrystal surfaces than can be achieved with the thinner 2ML shells.

The data reveal that the addition of the CdS shells to the 2 ML and 6 ML CdSe/CdS QDs resulted in marked increases of the time scale of their excited state relaxation, which is consistent with prior observations in the literature[2]. The kinetics traces in **Figure S5** were fit with tri-exponential functions to quantify the average time constant describing the decays. The fit functions appear as dashed curves overlayed on the data and color coded to the experimental data. **Table S2** tabulates the best fit parameters and their uncertainty limits obtained from the least squares fitting procedure. The amplitude-weighted average time constants of the kinetic decays obtained from this procedure are 55 ± 10 ns, 300 ± 30 ns, and 530 ± 70 ns for the CdSe core-only, 2 ML CdSe/CdS, and 6 ML CdSe/CdS QD samples, respectively. The uncertainty limits of these average time constants were dominated by the uncertainty of the amplitudes of the longest decay components of the kinetics of each sample.

Table S2. Table of best fit parameters and uncertainty limits of 1S-1P electronic transitions. The parameters correspond to the tri-exponential fit functions used to quantify the intensity-weighted average decay times of the electronic transitions in the CdSe core-only, 2ML CdSe/CdS, and 6ML CdSe/CdS QD films.

	**CdSe cores**	**2ML CdSe/CdS**	**6ML CdSe/CdS**
τ1 (ns)	15 $\pm$ 5	27 $\pm$ 7	30 $\pm$ 7
A1	0.75 $\pm$ 0.1	0.67 $\pm$ 0.1	0.47 $\pm$ 0.05
τ2 (ns)	110 $\pm$ 10	280 $\pm$ 20	300 $\pm$ 50
A2	0.22 $\pm$ 0.05	0.24 $\pm$ 0.03	0.32 $\pm$ 0.05
τ3 (ns)	610 $\pm$ 100	2500 $\pm$ 200	2000 $\pm$ 300
A3	0.03 $\pm$ 0.01	0.09 $\pm$ 0.02	0.21 $\pm$ 0.04
Ave. Lifetime	55 $\pm$ 10	300 $\pm$ 30	530 $\pm$ 70

Table S3. Table of best fit parameters and uncertainty limits of Fano-type vibrational transitions. The parameters correspond to the tri-exponential fit functions used to quantify the intensity-weighted average decay times of the Fano-type vibrational features of the ligand carboxylic acid groups of CdSe core-only, 2ML CdSe/CdS, and 6ML CdSe/CdS QD films.

	**CdSe cores**	**2ML CdSe/CdS**	**6ML CdSe/CdS**
τ1 (ns)	15 $\pm$ 5	35 $\pm$ 7	30 $\pm$ 7
A1	0.6 $\pm$ 0.1	0.53 $\pm$ 0.1	0.43 $\pm$ 0.05
τ2 (ns)	110 $\pm$ 10	290 $\pm$ 20	300 $\pm$ 50
A2	0.36 $\pm$ 0.05	0.33 $\pm$ 0.03	0.34 $\pm$ 0.05
τ3 (ns)	610 $\pm$ 100	2500 $\pm$ 200	2000 $\pm$ 300
A3	0.04 $\pm$ 0.01	0.14 $\pm$ 0.02	0.23 $\pm$ 0.04
Ave. Lifetime	70 $\pm$ 15	460 $\pm$ 50	570 $\pm$ 70

<u>Section S8</u>: Literature Cited.

(1) Lv, L.; Li, J.; Wang, Y.; Shy, Y.; Peng, X., Monodisperse Cdse Quantum Dots Encased in Six (100) Facets Via Ligand-Controlled Nucleation and Growth. *J. Am. Chem. Soc.* **2020**, *142*, 19926-19935.
(2) Cerillo, M.; Aubert, T.; Gomes, R.; Van Duen, V.; Emplit, P.; Biermann, A.; Lange, H.; Thomson, C.; Brainis, E.; Hens, Z., "Flash" Synthesis of Cdse/Cds Core-Shell Quantum Dots. *Chem. Mater.* **2014**, *26*, 1154–1160.
(3) Wuister, S. F.; De Mello Donega, C.; Meijerink, A., Influence of Thiol Capping on the Exciton Luminescence and Decay Kinetics of Cdte and Cdse Quamtum Dots. *J. Phys. Chem. B* **2004**, *108*, 17393-17397.
(4) Buckley, J. J.; Couderc, E.; Greaney, M. J.; Munteanu, J.; Riche, C. T.; Bradforth, S. E.; Brutchey, R. L., Chalcogenol Ligand Toolbox for Cdse Nanocrystals and Their Influence on Exciton Relaxation Pathways. *ACS Nano* **2014**, *8*, 2512-2521.
(5) Yi, C.; Knappenberger, K. L., The Influence of Surface Passivation on Electronic Energy Relaxation Dynamics of Cdse and Cdse/Cds Nanocrystals Studied Using Visible and near Infrared Transient Absorption Spectroscopy. *Nanoscale.* **2015**, *7*, 5884-5891.
(6) Grega, M. N.; Cho, J. A.; Brown, R. A.; Asbury, J. B., Dynamic Nanocrystal-Ligand Boundaries: Reversible Photoinduced Ligand Detachment from Quantum Dots in Solution. *J. Am. Chem. Soc.* **2026**, *148*, 3501-3512.
